# Identification of Novel Potential Genes Involved in Cancer by Integrated Comparative Analyses

**DOI:** 10.3390/ijms21249560

**Published:** 2020-12-15

**Authors:** Francesco Monticolo, Emanuela Palomba, Maria Luisa Chiusano

**Affiliations:** 1 Department of Agricultural Sciences, Università Degli Studi di Napoli Federico II, 80055 Naples, Italy; f.monticolo90@gmail.com; 2 Department of RIMAR, Stazione Zoologica “Anton Dohrn”, 80122 Naples, Italy; ema.palomba91@gmail.com

**Keywords:** co-expression analysis, programmed cell death, autophagy, bioinformatics, conserved expression patterns

## Abstract

The main hallmarks of cancer diseases are the evasion of programmed cell death, uncontrolled cell division, and the ability to invade adjacent tissues. The explosion of omics technologies offers challenging opportunities to identify molecular agents and processes that may play relevant roles in cancer. They can support comparative investigations, in one or multiple experiments, exploiting evidence from one or multiple species. Here, we analyzed gene expression data from induction of programmed cell death and stress response in *Homo sapiens* and compared the results with *Saccharomyces cerevisiae* gene expression during the response to cell death. The aim was to identify conserved candidate genes associated with *Homo sapiens* cell death, favored by crosslinks based on orthology relationships between the two species. We identified differentially-expressed genes, pathways that are significantly dysregulated across treatments, and characterized genes among those involved in induced cell death. We investigated on co-expression patterns and identified novel genes that were not expected to be associated with death pathways, that have a conserved pattern of expression between the two species. Finally, we analyzed the resulting list by HumanNet and identified new genes predicted to be involved in cancer. The data integration and the comparative approach between distantly-related reference species that were here exploited pave the way to novel discoveries in cancer therapy and also contribute to detect conserved genes potentially involved in programmed cell death.

## 1. Introduction

Cancer is the second leading cause of death in the world. It was responsible for 9,6 million deaths in 2018 (https://www.who.int/news-room/fact-sheets/detail/cancer) and in 2020, it is expected to be the cause of 606,520 deaths in the United States [1] and 1.3 million deaths in the Europe (https://ecis.jrc.ec.europa.eu/). Cancer hallmarks include six biological aspects—sustaining proliferative signaling, evading growth suppressors, enabling replicative immortality, inducing angiogenesis, activating cell invasion and metastasis, and resisting to programmed cell death (PCD) [2]. PCD includes heterogeneous processes that are triggered by development or stress [3]. Therefore, the study of pathways associated with PCD is crucial in the field.

In *Homo sapiens*, 16 different types of PCD are currently described—apoptosis (intrinsic and extrinsic) [4,5,6], mitotic death [3], mitochondrial permeability transition driven necrosis [7], necroptosis [8], pyroptosis [9], ferroptosis [10], lysosome-dependent cell death [11], parthanatos [12], entosis [13], autophagy-related cell death [14], autosis [15], immunogenic cell death [16], NETotic cell death [17], apoNETosis [18], oxeiptosis [19], and alkaliptosis [20].

Apoptosis is the best-characterized programmed cell death in *H. sapiens,* where it can also contribute to physiological phenomena like embryogenesis [21]. It is induced by both intrinsic (DNA damage, replication stress, endoplasmic reticulum stress, reactive oxygen species (ROS), and microtubule alterations) and extrinsic stimuli (initiated by perturbations of the extracellular microenvironment) [3].

Knowledge of the mechanisms involved in the process of apoptosis in mammalian cells comes from the study of PCD the development of the nematode *Caenorhabditis elegans* [22]. Apoptosis in *C. elegans* occurs during two stages and in two different tissues—embryonic and post-embryonic development of soma and in the gonad of adult hermaphrodites [23,24]. Once the apoptotic program is activated, by the alteration of mitochondrial permeability that causes the release of mitochondrial pro-apoptotic content into the cytoplasm [25], it leads to nuclear DNA fragmentation, cytoplasm shrinkage, and phosphatidylserine flipping onto the outer plasma membrane to induce phagocytosis by neighboring cells [26]. Phagocytosis is essential for removing cell corpses generated by PCD [27].

PCD frequently occurs with, or is preceded by, autophagy. Autophagy is a lysosome-mediated degradation pathway essential for survival, differentiation, development, and homeostasis [28]. Three types of autophagy are currently described—chaperone-mediated, microautophagy, and macroautophagy [29]. Chaperone-mediated autophagy, that requires chaperone proteins, involves the direct translocation of cytosolic proteins into the lysosome. Microautophagy transports a small portion of cytoplasm into the lysosome through the invagination of the lysosomal membrane. Macroautophagy is mediated by the autophagosome. The autophagosome is an organelle formed by a double membrane. Starting from a small vesicle, called phagophore, the elongation and the inclusion of a portion of cytoplasm leads to the formation of the autophagosome. Subsequently, the outer membrane of the autophagosome fuses with a lysosome, leading to the degradation of the portion of the cytoplasm included into the autophagosome. All the small molecules generated by this degradation are released to the cytoplasm contributing to recycling and/or energy production [29]. In some cases, autophagic membranes facilitate the activation of apoptotic cell death [30].

As PCD dysfunctions are associated with tumorigenesis, several genetic links have also emerged between autophagy dysfunctions and cancer [28,31,32,33,34,35], thanks to the initial discovery by Liang et al. in 1999 [31], demonstrating that *BECLIN1* (a key regulator of autophagy) is a candidate tumor suppressor [31].

Autophagy and PCD are processes widely described not only in *H. sapiens* but also in *Saccharomyces cerevisiae* (yeast) [36,37].

Yeast PCD can be triggered by drugs, heat stress, UV radiation, heterologous expression of pro-apoptotic human genes, and acetic acid [38,39], determining canonical apoptosis hallmarks such as chromatin condensation, DNA fragmentation, dissipation of the mitochondrial transmembrane potential, and mitochondria cytochrome c (CYT-*c*) release [40]. *S. cerevisiae* is also a model species for the study of autophagy. Indeed, the autophagy-related proteins (ATGs), specifically involved in autophagy, were first described in yeast [41]. In addition, this species also possesses all the principal signaling cascades that are also present in *H. sapiens* and modulate the cell metabolism in response to nutrient availability [42].

Our approach focused on a collection of *H. sapiens* transcriptome data based on RNA-seq experiments associated with PCD. It identified dysregulated pathways across the experiments and determined co-expressed genes, to expand the list of potential novel human genes involved in PCD. The overall list of co-expressed genes that we derived from the analysis of nine experiments associated with PCD was then crosschecked with transcriptome expression levels from 27 experiments of human cell stress responses, to identify key genes associated with exclusive patterns typical of cell-death response.

We compared the list of human candidates with the corresponding cell-death-related ortholog genes in *S. cerevisiae*, to identify potential conserved markers of cell death and novel targets for cancer therapy. Lastly, a preliminary investigation of the resulting conserved genes and their possible role was done by exploiting HumanNet [43], to infer on their possible involvement in human pathologies. Thirteen genes were found to be already associated with cancer diseases, three were not reported as involved or were not predicted in pathologies, while other 16 genes were predicted here for the first time to be involved in cancer.

## 2. Results

**Figure 1:**
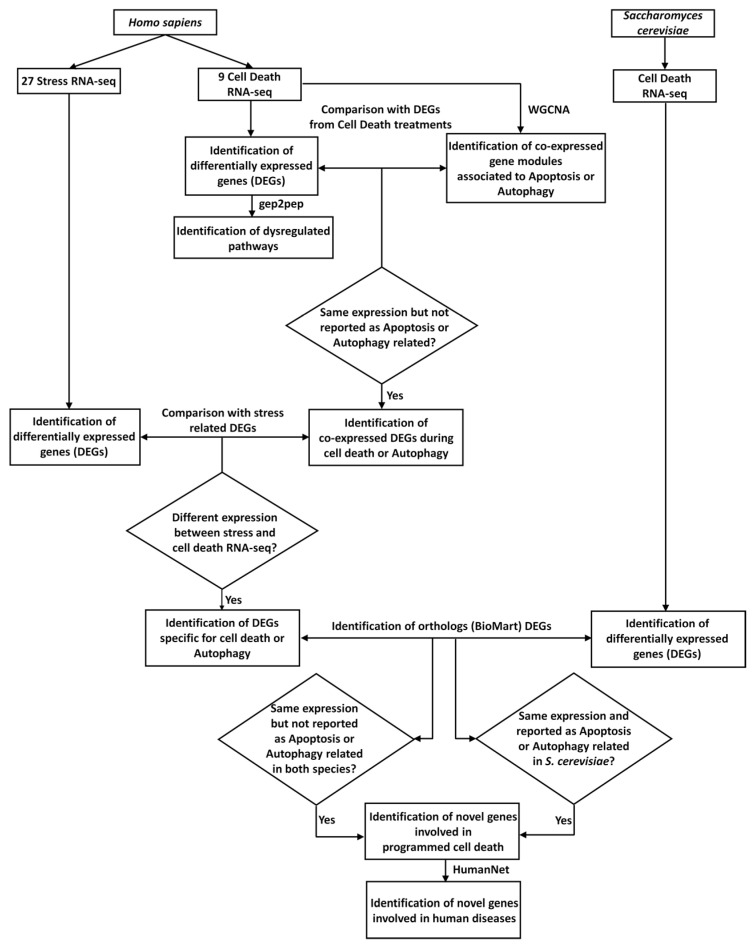
Scheme of the analytical procedure here proposed. Collection of RNA-seq experiments related to cell death (nine cell death RNA-seq) and stress (27 Stress RNA-seq), for *Homo sapiens,* and related to cell death, for *Saccharomyces cerevisiae* (cell death RNA-seq), respectively. Differentially-expressed genes (DEGs) obtained from cell-death-related RNA-seq (identification of differentially-expressed genes (DEGs)) were used as for the identification of dysregulated pathways, using gep2pep (identification of dysregulated pathways). Normalized mapped reads related to cell death in *H. sapiens* were used for the co-expression analysis, using Weighted Correlation Network Analysis (WGCNA) (identification of co-expressed gene modules associated with apoptosis or autophagy). DEGs with the same expression pattern in the nine cell death RNA-seq experiments were compared with modules of co-expressed genes enriched in gene ontologies (GO) or pathways related to apoptosis and/or autophagy. DEGs with the same expression pattern, and not reported as apoptosis- or autophagy-related, were considered (identification of co-expressed DEGs during cell death). Subsequently, these DEGs were compared with stress-related DEGs, to identify DEGs specific to cell death (identification of DEGs specific for cell death). Independently, DEGs related to cell death for *S. cerevisiae* were also identified. Through the orthology relationships between *H. sapiens* and *S. cerevisiae* (downloaded from BioMart), DEGs specific for human cell death were compared with those from *S. cerevisiae* cell death. This analysis led to the identification of genes involved in cell death with conserved functionalities in the two species (identification of novel genes involved in PCD). Lastly, using HumanNet [43] the genes predicted to be involved in cell death were investigated for their involvement in human diseases (identification of novel genes involved in human diseases).

The analytical procedure exploited in this work is summarized in Figure 1.

### 2.1. H. sapiens Cell-Death-Related Differentially-Expressed Genes and Dysregulated Pathways

**Figure 2:**
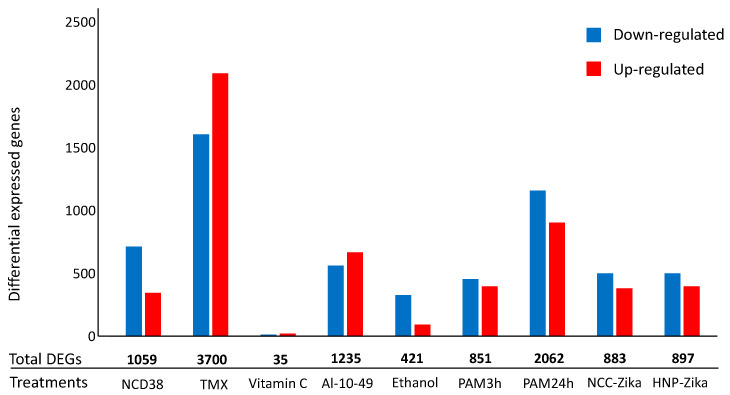
Number of differential expressed genes (DEGs) in *H. sapiens* cell-death-related RNA-seq experiments. The histogram represents the number of up-regulated (red) and down-regulated (blue) DEGs for each experiment. Numbers in bold represent the total number of DEGs per experiments.

The number of differentially expressed genes (DEGs) resulting from the processing of the nine RNA-seq experiments on *H. sapiens* cells exposed to apoptotic stimuli (Table S1) are reported in Figure 2.

DEG analysis by the gep2pep package [44] highlighted 31 significantly dysregulated pathways, among which two pathways were strictly associated with gene ontology (GO) terms associated with apoptosis and/or to autophagy, both enriched by up-regulated genes (Table 1).

Interestingly, among the 7420 DEGs in at least one treatment, 2548 were annotated as apoptosis- and/or autophagy-related genes, considering the GO or the Kyoto Encyclopedia of Genes and Genomes (KEGG) annotations. Among the 2548 genes, considering those that had the same expression pattern in at least two different treatments among the nine cell death experiments here considered, 822 and 131 genes were the DEGs associated exclusively with apoptosis or to autophagy, respectively, while 75 genes had both the keywords in their annotations (Table S2). Despite the heterogeneity of the considered treatments (Figure 2), among these 1028 DEGs, the up-regulated DEGs were 612 and those present in at least five experiments (from five to eight) were 27. Moreover, the down-regulated DEGs were 416 and those present in at least five experiments were three. In addition, among the 1028 genes, 103 had orthologs in yeast and 19 show the same expression patterns in the human- and yeast-related data in cell death experiments (a total of 12 datasets (Table S2)). Interestingly, the range of experiments with common expression patterns for the 19 genes went from two to six.

### 2.2. H. sapiens Co-Expressed Genes in Cell-Death-Related RNA-Seq Experiments

In order to identify genes involved in PCD, we performed a co-expression network analysis using Weighted Correlation Network Analysis (WGCNA) [45], that resulted in 20 clusters of co-expressed genes in the treatments (Figure S1, colored modules), and 18 clusters in the controls (Figure S2). Permutating the 20 modules concerning the treatments, all demonstrated high preservation with Zsummary statistic >10 (Figure S3).

The GO enrichment analysis of the clusters in both treatment and control experiments resulted in two modules (the blue and light yellow modules), that were enriched in GO terms related to cell death processes only in treatment experiments (Table S3). Moreover, performing a functional enrichment analysis, two modules (the pink and midnight blue modules), together with the confirmation of one of the modules confirmed by the GO analysis (the blue module), resulted in the enrichment with genes related to apoptosis (Table S4). Moreover, two modules (the magenta and turquoise modules) were enriched with genes related to autophagy (Table S4).

**Figure 3:**
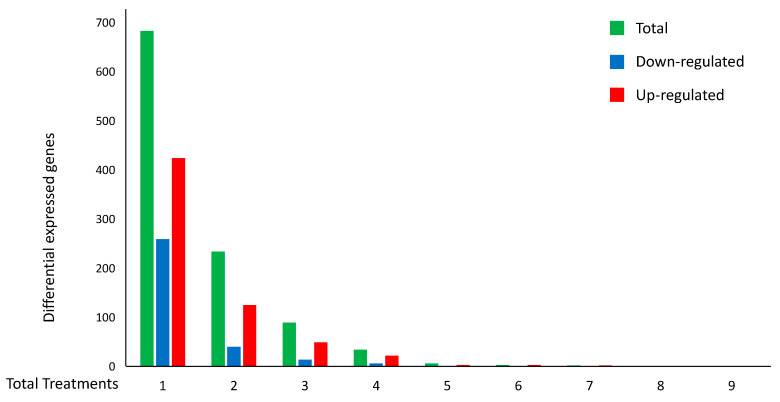
Distributions of the number of DEGs per number of *H. sapiens* cell-death-related experiments. The histogram represents the total number of DEGs (green) for cell-death treatments. The number of up-regulated (red) and down-regulated (blue) DEGs with the same expression pattern are also reported.

The six modules of co-expressed genes in the treatments contain a total of 3180 genes, 1560 genes in modules enriched with gene ontology terms and pathways related to apoptosis, and 1620 genes in modules related to autophagy pathways, respectively. Interestingly, 2451 out of the 3180 genes were not annotated in either gene ontology categories [46] and/or to KEGG pathways [47] that could be associated with autophagy or apoptosis processes. Moreover, these genes were not reported as confirmed or potential genes involved in cancer, in the Catalogue Of Somatic Mutations In Cancer (COSMIC) database [48]. Furthermore, 1051 out of the 2451 genes that were identified by the co-expressed module analysis were confirmed to be DEGs in at least one of the experiments related to cell death, with 368 being DEGs in more than one experiment (Table 2 and Figure 3).

In particular, Figure 3 and Table 2 also summarize the distribution of the 948 concordant genes, i.e., those genes with the same expression pattern (always up- or down-regulated in the different experiments (red or blue, respectively)). Interestingly, among the 948 concordant genes, 265 were confirmed as DEGs in at least two experiments. On the other hand, the maximum number of experiments with concordant DEGs was seven, and specifically, they were up-regulated genes.

The comparison of the expression profiles of the 948 concordant DEGs from death-related experiments with their expression in stress-related experiments (Table S1) helped to define the list of 734 genes expressed with an exclusive or specific pattern in the cell-death experiments. They included those that were DEGs only in cell-death-related experiments, and those with opposite expression patterns in comparison with stress experiments (Table 2). In particular, 655 out of 734 DEGs were expressed exclusively in cell death, while the remaining 79 DEGs had an opposite behavior in stress-related experiments.

### 2.3. Cross-Comparisons between H. sapiens and S. cerevisiae Gene-Expression Profiles in Cell Death and Prediction of Disease-Related Genes.

In the list of 734 human DEGs, 278 genes have orthologs in *S. cerevisiae*. We compared their expression profiles with the ones of the corresponding best orthologs in *S. cerevisiae* in response to acetic acid exposure, which notoriously determines PCD in yeast [49]. Interestingly, 32 *H. sapiens* DEGs had an ortholog that resulted to be a DEG in *S. cerevisiae*, and they showed the same expression pattern, either up- or down-regulated (Table 2 and Table S5). Interestingly, two out of 32 genes had the corresponding *S. cerevisiae* orthologs that were endowed by a GO associated with apoptosis or autophagy, while the human genes were not (Table 2 and Table S5).

A preliminary investigation of the 32 *H. sapiens* genes with conserved patterns with orthologs in *S. cerevisiae* was performed exploiting the HumanNet tool (http://www.functionalnet.org/humannet/), to predict their role as potential disease-related genes [43]. The tool permits confirmation or prediction of the association of a gene with a disease, by analyzing functionally-annotated networks of its neighborhoods [43], and inferring the probability that connected genes have to trigger a common disease. The disease annotation prediction analysis gathers information from the neighboring genes in the network exploiting different resources (DisGeNET [50], DISEASES [51], Disease Ontology Annotation Framework (DOAF) [52], Human Phenotype Ontology (HPO), and GO biological process), and prioritizes candidate disease genes by significance levels [43]. While this tool confirmed the involvement of 16 among the 32 genes here detected with cancer, interestingly, the other 16 genes were predicted (using Bayesian statistics) to be associated with this type of disease, too, although the genes have not yet been associated with this pathology (Table S6).

## 3. Discussion

*S. cerevisiae* has been always considered a model species to confirm *H. sapiens* gene functions, especially in cell-death-related studies [53,54,55]. Here, we describe a comparative integrated approach that helped to expand the list of candidate genes involved in cell death in *Homo sapiens.* These genes have orthologs with the same expression pattern also in *S. cerevisiae*, paving the way to the discovery of novel conserved pathways between distantly-related reference species investigating on conserved gene functionalities in cell death.

Specifically, we report results on whole transcriptome analysis from nine human cell-death-related RNA-seq experiments. Our results highlight that the activation of PCD induces heterogeneous transcriptomic changes (Figure 2). They also show that transcriptomic changes are associated with pathways related to the intrinsic apoptotic pathway and to the autophagy of mitochondrion (mitophagy) (Table 1). It is well-known that mitophagy functions as an early protective response, favoring adaptation to stress by removing damaged mitochondria [56]. In contrast, increased oxidative stress and apoptotic proteases can overcome mitophagy, causing the activation and progress of cell death [57]. In response to stress, pro-survival and pro-death pathways are concomitantly activated and the final outcome depends on a complex crosstalk between these pathways, determining cell fate [58].

Specifically, we identified 7420 DEGs. Of these, 1028 genes are reported as involved in apoptosis and/or autophagy, and are DEGs with the same expression pattern in at least two different treatments among the nine cell-death-related RNA-seq experiments. Among these 1028 DEGS, 35 are DEGs in at least five of the nine treatments.

Among these, the gene *PPP1R15A* (*ENSG00000087074*) is up-regulated in 8 treatments (the only exception is the treatment with vitamin C). PPP1R15A is involved in apoptosis following ionizing radiation [59], methyl-methanesulfonate treatments [60], and in endoplasmic reticulum stress [58]. In 2020, Gambardella et al. [61] found that PPP1R15A also plays an essential role in autophagy, by enabling lysosomal biogenesis and a sustained autophagic flux [61]. In fact, PPP1R15A is also essential for translation during starvation [61] but also in apoptosis, if the endoplasmic reticulum stress is not resolved [62]. Therefore, PPP1R15A plays a crucial role in both autophagy and apoptosis.

*DDIT3* (*ENSG00000175197*) represents another example. It is up-regulated in six treatments. *DDIT3* is expressed during endoplasmic reticulum stress and can stimulate autophagosome formation through down-regulation of *BCL2* (*ENSG00000171791*) (that is down-regulated in four treatments). Autophagy induction that relies on the transcription activity of DDIT3 reflects a cytotoxic condition that occurs only during the final stage of the endoplasmic reticulum stress [63]. Interestingly, the transcriptional activity of DDIT3 increases cell death [64]. In fact, the over-expression of *DDIT3* sensitizes cells to apoptosis through the down-regulation of *BCL2*, the depletion of cellular glutathione, and the production of ROS [65].

The gene *SKP2* (*ENSG00000145604*) is down-regulated in five treatments. In 2005, Harada et al. [66] found that the down-regulation of *SKP2* appears to induce apoptosis in oral cancer cells. In fact, Skp2-anti-sense treatments induce apoptosis characterized by an increase in the fragmentation of nuclei and activation of caspases [66]. Moreover, the growth of xenograft tumors is also markedly suppressed by Skp2-anti-sense treatment [66].

Using orthology relationships between *H. sapiens* and *S. cerevisiae,* we identified 19 genes among the 103 orthologs genes of the 1028 DEGs that had the same expression pattern in *H. sapiens* and *S. cerevisiae* cell-death treatments. These genes can represent conserved functionalities in apoptosis or autophagy related genes and deserve further investigations. Among these genes, *HMOX1* is up-regulated in 5 human treatments and in 1 yeast treatment. HMOX1 is considered to exert antiapoptotic and cytoprotective effects [67], but these results need further investigations to elucidate the exact mechanisms of action.

Another example is *PLK1* (*ENSG00000166851*), that is down-regulated in 2 *H. sapiens* cell-death treatments while its ortholog, *Cdc5* (*Ymr001c*), is down-regulated in one of the stages in the yeast cell-death treatment. *PLK1* was found to be up-regulated in many different types of cancer, in fact, it has been proposed as a novel diagnostic marker for several tumors [68]. In 2003, Liu et al. [68] showed that PLK1 depletion dramatically inhibited cell proliferation, decreased viability, and resulted in cell-cycle arrest [68]. These two examples reveal the potential outcomes of analyses of shared DEGs among different human experiments, and, in addition, of cross-comparisons with expression patterns of yeast orthologs.

Our analysis of human cell-death-related transcriptomes also highlighted six modules of co-expressed genes, including a total of 3180 genes, that are enriched in GO related to apoptosis or autophagy related pathways. Interestingly, 2451 out of 3180 genes are not reported with a GO [46] or in a pathway [47] related to cell-death and/or autophagy processes, neither they are reported as genes involved in cancer [48]. Moreover, 1051 out of 2451 genes are DEGs in at least one experiment related to cell death and 734 are DEGs with typical expression patterns exclusive to cell death when comparing the results with 27 experiments from human-cell stress response. Noticeably, 24 out of 734 DEGs are reported as novel proteins or transcripts. Interestingly, 173 have confirmed expression patterns in more than two experiments of cell death and four are confirmed in at least five of nine experiments.

Comparing the 734 *H. sapiens* cell-death-specific DEGs with their ortholog DEGs in *S. cerevisiae* exposed to acetic acid [49], we confirmed 32 DEGs with the same expression patterns.

These genes can be considered as conserved candidate genes involved in cell death in both species. We analyzed these genes as candidates in human diseases. Interestingly we found 16 genes that are predicted to be involved and that are not yet associated with cancer by the HumanNet tool (Table S6). Among these 16 genes predicted to be involved in cancer, we will highlight some examples. *ABDH11* (*ENSG00000106077*) in *H. sapiens* is an ortholog with the yeast gene *Imo32* (*Ygr031w*), and both genes are down-regulated in the two species. In 2020, Bayley et al. [69] demonstrated that loss or inhibition of ABHD11 leads to rapid increase in 2-oxoglutarate levels [69]. High levels of 2-oxoglutarate leads to reduced tumor growth, and this indicates that an increase of 2-oxoglutarate levels may contribute to a possible anti-cancer effect [70].

The human gene *SURF1* (*ENSG00000148290*) and its yeast ortholog, *Shy1* (*Ygr112w*), are both up-regulated in the two species. *SURF1*, encoding a factor involved in the biogenesis of cytochrome c oxidase, is associated with the Leigh syndrome, a mitochondrial disorder caused by mutations of *SURF1*. In 2007, D’Agnello et al. [71] showed that adult SURF1−/− mice were protected from neurodegeneration at any age, showing prolonged lifespan and complete protection from Ca^2+^-dependent neurotoxicity induced by kainic acid [71].

The human gene *LTV1* (*ENSG00000135521*) and its yeast ortholog *Ltv1* (Ykl143w) are up-regulated genes in both species. In yeast, the ribosome assembly factor Ltv1p facilitates the incorporation of Rps3p, Rps10p, and Asc1p/RACK1 into the small ribosomal subunit. Ribosomes from Ltv1-deficient yeasts have reduced amounts of Rps10p and Asc1p and show defects in translational fidelity and ribosome-mediated RNA quality control. These defects provide a growth advantage, but sensitize the cells to oxidative stress. Interestingly, glioma cancer cells have reduced levels of LTV1 and produce ribosomes lacking RPS3, RPS10, and RACK1 [72].

Our preliminary results on the 16 genes identified as candidate cell-death-related genes in both *H. sapiens* and *S. cerevisiae*, possibly involved in cancer, reveal the opportunities that similar comparative approaches can have in depicting novel programmed cell-death-related genes in both species, and pave the way to further computational and/or experimental validations to assign their role to novel pathways and/or consider their usefulness as targets for anti-cancer therapy.

## 4. Materials and Methods

RNA-seq data (fastq files) from nine experiments on *Homo sapiens* apoptosis [73,74,75,76,77,78,79] and 36 from stress conditions [80,81,82,83,84,85] (Table S1) together with one experiment (SRP075510) on expression profiling at three different time points from *Saccharomyces cerevisiae* induced cell death by exposure to acetic acid [49], plus all control data, were downloaded from sequence read archive (SRA) [86].

Raw reads were trimmed with Trimmomatic (release 0.38) [87] and mapped onto the respective reference genomes (*Homo sapiens* genome sequence (version GRCh38.96) and *Saccharomyces cerevisiae* genome (strain S288c, version R64-1-1)), using STAR (release 2.6.0) [88] (settings: outFilterScoreMinOverLread = 0, outFilterMatchNminOverLread = 0, outFilterMatchNmin = 0, alignIntronMax = 10,000, and other parameters as default values). Read counting was performed using FeatureCounts (release 1.6.3) [89] (settings: t = “exon”, g = “gene_id”, s = “0”, with the overlapping option and other parameters as default values). Read counts were normalized by counts per million (CPM), filtering protein coding genes with CPM ≥ 1 in each replicate per treatment and control. The differential expression analysis was done using edgeR [90]. Only genes with |log_2_Fold change| ≥ 1 and FDR < 0.05 were considered as significant differentially-expressed genes (DEGs).

Dysregulated pathways, i.e., pathways significantly involving differentially-regulated genes across the experiments, were obtained using gep2pep (v 1.8.0) [44] using as pathway database MSigDB (v 7.1) [91] and only pathways with false discovery rate (FDR) < 0.01 were considered as significant.

Co-expression analysis to identify different modules in *Homo sapiens* apoptosis was performed with the R package WGCNA [45], using as input the log2(reads per kilobase million (RPKM) + 1) of protein-coding genes with at least 10 raw reads mapped in at least 80% of replicates. The parameters used are listed in Table S7.

To test whether the identified modules are stable, the modulePreservation function of WGCNA package [45] (with nPermutations = 200) was used. In input log2(RPKM + 1) of protein coding genes with at least 10 raw reads mapped in at least 80% of the replicates of apoptotic treatments were considered. The preservation statistic Zsummary was used to quantify the preservation of gene modules among the datasets.

*Homo sapiens* gene ontology (GO) enrichment analysis was performed using the anRichment R package [92], filtering enriched GOs at FDR < 0.01.

Enriched biochemical pathways were obtained by the ConsensusPathDB (http://cpdb.molgen.mpg.de/CPDB) [93], filtering enriched pathways at *q*-value < 0.01.

GO annotation and orthologs between *Homo sapiens* and *Saccharomyces cerevisiae* were obtained from Biomart (Ensembl release 101) (https://m.ensembl.org/info/data/biomart/index.html) [46].

Genes involved in cancer pathways were obtained from COSMIC (v92) [48] and KEGG databases (release 95.2) [47]. Genes involved in autophagy were obtained from the human autophagy database (http://autophagy.lu/clustering/).

## Figures and Tables

**Table 1:** List of dysregulated pathways of *H. sapiens* resulting from the differential expression analysis of the cell-death-related treatments. Significant dysregulated pathways, relative enrichment score (ES), and corresponding *p*-value (PV) in cell-death treatments. Dysregulated pathways associated with cell death are highlighted in bold.

Dysregulated Pathways	ES	PV
**Defense response to other organism**	1	0.001
Innate immune response	1	0.001
**Cellular response to topologically-incorrect protein**	1	0.002
**Regulation of response to biotic stimulus**	1	0.002
**Response to topologically-incorrect protein**	1	0.002
**FC epsilon receptor-signaling pathway**	1	0.003
**Natural killer cell-mediated immunity**	1	0.003
Golgi organization	1	0.003
**Autophagy of mitochondrion**	1	0.003
**Negative regulation of innate immune response**	1	0.004
Protein-k48-linked ubiquitination	1	0.004
Response to cytokine	0.889	0.006
**Tumor necrosis-factor-mediated signaling pathway**	0.875	0.009
**Intrinsic apoptotic signaling pathway**	0.875	0.009
**Positive regulation of response to biotic stimulus**	0.875	0.009
Organelle disassembly	0.875	0.009
**Endoplasmic reticulum unfolded-protein response**	0.875	0.009
Forebrain development	−0.875	0.009
Embryonic morphogenesis	−0.875	0.009
Head development	−0.875	0.009
Tube formation	−0.875	0.009
Urogenital-system development	−0.875	0.009
Homophilic-cell adhesion via plasma-membrane-adhesion molecules	−0.875	0.009
**Reactive-oxygen-species metabolic process**	−0.875	0.009
Male sex differentiation	−0.875	0.009
**Development of primary sexual characteristics**	−0.875	0.009
Regulation of neurotransmitter levels	−0.889	0.006
Osteoclast differentiation	−1	0.002
Spindle organization	−1	0.003
**Regulation of membrane-lipid distribution**	−1	0.008
Phospholipid transport	−1	0.008

**Table 2:** Number of *H. sapiens* cell-death candidate DEGs. Numbers based on the analysis of the orthologs with *S. cerevisiae* cell-death DEGs are included. The number of *H. sapiens* cell death candidate DEGs (cell-death DEGs), of the DEGs that had the same pattern of expression in *H. sapiens* cell death experiments (concordant cell-death DEGs), the concordant DEGs of *H. sapiens* with expression patterns specific to cell death (concordant DEGs specific to cell death versus stress), the number of orthologs with *S. cerevisiae*, their number when compared with *S. cerevisiae* DEGs having a GO related to cell death or autophagy (confirmed in *S. cerevisiae* by GO), and the number when considering *S. cerevisiae* DEGs with the same expression patterns (concordant in *S. cerevisiae* cell death), are all reported.

Occurrence in Cell-Death Treatments	Cell-Death DEGs	Concordant Cell-Death DEGs	Concordant DEGs Specific to Cell Death versus Stress	Orthologs with *S. cerevisiae*	Confirmed in *S. cerevisiae* by GOs	Concordant in *S. cerevisiae* Cell Death
**1**	683	683	561	155	2	27
**>1**	368	265	173	61	0	5
**Total**	1051	948	734	216	2	32

## Supplementary Materials

The following are available online at https://www.mdpi.com/1422-0067/21/24/9560/s1: Figure S1, Dendrogram of WGCNA modules of cell-death-related treatments; Figure S2, Dendrogram of WGCNA modules of controls; Figure S3, medianRank and Zsummary statistics of modules in module preservation; Table S1, List of RNA-seq datasets; Table S2, *H. sapiens* DEGs with same expression pattern related to apoptosis or autophagy and conserved in *S. cerevisiae;* Table S3, Gene ontology enrichment of WGCNA modules; Table S4, Pathways enrichment; Table S5, List of *H. sapiens* cell-death DEGs and *S. cerevisiae* orthologs and their expression during cell death; Table S6, HumanNet current knowledge or disease prediction for the 32 *H. sapiens*–*S. cerevisiae* ortholog genes; Table S7, Parameters used for WGCNA.
